# Сравнение эффективности различных методов определения уровня метанефринов в диагностике феохромоцитом

**DOI:** 10.14341/probl13309

**Published:** 2023-10-16

**Authors:** Ш. Ш. Шихмагомедов, Д. В. Реброва, Л. М. Краснов, Е. А. Федоров, И. К. Чинчук, Р. А. Черников, В. Ф. Русаков, И. В. Слепцов, Е. А. Згода

**Affiliations:** Санкт-Петербургский государственный университет, Клиника высоких медицинских технологий им. Н.И. Пирогова; Санкт-Петербургский государственный университет, Клиника высоких медицинских технологий им. Н.И. Пирогова; Санкт-Петербургский государственный университет, Клиника высоких медицинских технологий им. Н.И. Пирогова; Санкт-Петербургский государственный университет, Клиника высоких медицинских технологий им. Н.И. Пирогова; Санкт-Петербургский государственный университет, Клиника высоких медицинских технологий им. Н.И. Пирогова; Санкт-Петербургский государственный университет, Клиника высоких медицинских технологий им. Н.И. Пирогова; Санкт-Петербургский государственный университет, Клиника высоких медицинских технологий им. Н.И. Пирогова; Санкт-Петербургский государственный университет, Клиника высоких медицинских технологий им. Н.И. Пирогова; Санкт-Петербургский государственный университет, Клиника высоких медицинских технологий им. Н.И. Пирогова

**Keywords:** метанефрины, феохромоцитома, новообразования надпочечников

## Abstract

**ОБОСНОВАНИЕ:**

ОБОСНОВАНИЕ. Феохромоцитома (ФХЦ) — опухоль из хромаффинной ткани мозгового вещества надпочечника, способная к гиперпродукции катехоламинов. Повышенная продукция гормонов опухолью приводит к катехоламиновым кризам, оказывающим патологическое влияние на все органы и системы. В первичной диагностике феохромоцитом важное значение имеет определение уровня метаболитов катехоламинов — метанефринов. В настоящее время в клинической практике применяются различные методы определения уровня данного метаболита: в плазме крови или в моче, общего количества или только свободной формы, фракционированный анализ или нефракционированный.

**ЦЕЛЬ:**

ЦЕЛЬ. Сравнение эффективности различных методов определения уровня метанефринов для диагностики феохромоцитом.

**МАТЕРИАЛЫ И МЕТОДЫ:**

МАТЕРИАЛЫ И МЕТОДЫ. Ретроспективное одноцентровое когортное исследование было проведено на выборке пациентов, первично прооперированных по поводу новообразования надпочечника в Клинике высоких медицинских технологий им. Н.И. Пирогова СПбГУ с ноября 2007 по декабрь 2022 гг. и сдававших анализы на определение уровня метанефринов крови или мочи перед оперативным лечением. Оценивались результаты анализов на метанефрины и размер опухоли.

**РЕЗУЛЬТАТЫ:**

РЕЗУЛЬТАТЫ. Исследованы 1088 пациентов с новообразованиями надпочечников, подвергшихся оперативному лечению, из которых у 348 было гистологически подтверждено наличие феохромоцитомы. Выполнено сравнение четырех видов анализов на метанефрины: свободные фракционированные метанефрины плазмы (232 пациента), нефракционированные метанефрины суточной мочи (431 пациент), фракционированные общие метанефрины суточной мочи (427 пациентов) и фракционированные свободные метанефрины суточной мочи (178 пациентов). Наибольшую чувствительность продемонстрировал анализ свободных фракционированных метанефринов плазмы крови (95,4%). В отличие от других, чувствительность данного анализа не снижалась в группе больных с феохромоцитомами небольших размеров (3 см и менее). Наибольшую специфичность продемонстрировал анализ нефракционированных метанефринов суточной мочи (97,8%), при наименьшей среди всех тестов чувствительности (67,6%). Исследование фракционированных общих метанефринов суточной мочи показало хорошие результаты чувствительности и специфичности, лишь немного уступая лучшим показателям, а анализ свободных метанефринов суточной мочи продемонстрировал неожиданно низкую эффективность. Между уровнем метанефринов в крови и размерами опухоли установлено наличие положительной корреляции.

**ЗАКЛЮЧЕНИЕ:**

ЗАКЛЮЧЕНИЕ. Исходя из полученных данных, предпочтительными анализами для первичной диагностики феохромоцитом можно назвать определение фракционированных свободных метанефринов плазмы и фракционированных общих метанефринов суточной мочи, что согласуется с существующими клиническими рекомендациями. Размер опухоли коррелирует с выраженностью повышения уровня метанефринов определенным любым из описанных способов.

## Abstract (en)

**BACKGROUND:**

BACKGROUND: Pheochromocytoma (PHEO) is a tumor from the chromaffin tissue of the adrenal medulla, capable of hyperproduction of catecholamines. The increased production of hormones by the tumor leads to catecholamine crises, which have a pathological effect on all organs and systems. In the primary diagnosis of pheochromocytomas, it is important to determine the level of the metabolite of catecholamines — metanephrines. Currently, in clinical practice, various methods are used to determine the level of this metabolite: in blood plasma or in urine, total or only free form, fractionated analysis or unfractionated.

**AIM:**

AIM: Comparison of the effectiveness of various methods for determining the level of metanephrines for the diagnosis of pheochromocytomas.

**MATERIALS AND METHODS:**

MATERIALS AND METHODS: A retrospective single-center cohort study was conducted on a sample of patients who were initially operated on for adrenal neoplasm at the Pirogov St. Petersburg State University High Medical Technology Clinic from November 2007 to December 2022 and who passed analysis to determine the level of blood or urine metanephrins before surgical treatment. The results of tests for metanephrine and tumor size were evaluated.

**RESULTS:**

RESULTS: 1088 patients with adrenal neoplasms who underwent surgical treatment were examined, of which 348 had histologically confirmed the presence of pheochromocytoma. Four types of metanephrine assays were compared: free fractionated plasma metanephrines (232 patients), unfractionated daily urine metanephrines (431 patients), fractionated total daily urine metanephrines (427 patients) and fractionated free daily urine metanephrines (178 patients). The greatest sensitivity was demonstrated by the analysis of free fractionated plasma methanephrines (95.4%). Unlike others, the sensitivity of this analysis did not decrease in the group of patients with small pheochromocytomas (3 cm or less). The greatest specificity was demonstrated by the analysis of unfractionated metanephrines in daily urine (97.8%), with the lowest sensitivity among all tests (67.6%). The study of fractionated total daily urine metanephrins showed good results of sensitivity and specificity, only slightly inferior to the best indicators, and the analysis of free daily urine metanephrins demonstrated unexpectedly low efficiency. There is a positive correlation between the level of metanephrine in the blood and the size of the tumor.

**CONCLUSION:**

CONCLUSION: Based on the data obtained, the preferred assays for the primary diagnosis of pheochromocytoma can be considered the determination of fractionated free plasma metanephrines and fractionated total daily urine metanephrines, which is consistent with relevant clinical recommendations. It was found that the size of the tumor correlates with the severity of an increase in the level of metanephrins determined by any of the described methods.

## ОБОСНОВАНИЕ

Феохромоцитома (ФХЦ) — опухоль из хромаффинной ткани мозгового слоя надпочечников, способная к гиперпродукции катехоламинов [1]. Повышенная продукция гормонов опухолью приводит к катехоламиновым кризам, оказывающим патологическое влияние на все органы и системы, с возможным развитием сердечно-сосудистых катастроф вплоть до летального исхода [2]. По классификации ВОЗ IV пересмотра от 2017 г., ФХЦ отнесена к злокачественным опухолям [3]. Важность ранней диагностики данных опухолей ассоциирована с выживаемостью пациентов в связи с риском развития опасной для жизни клинической симптоматики, а также риском инвазивного роста катехоламин-продуцирующих новообразований надпочечников и их метастазирования.

Знание о выделяемых опухолью гормонах привело к тому, что для диагностики ФХЦ на первых этапах использовался анализ крови и мочи на адреналин и норадреналин. Однако в дальнейшем было показано, что данный вариант лабораторной диагностики эффективен преимущественно во время приступа, ассоциированного с выбросом ФХЦ катехоламинов в кровь. В то же время в межприступный период высок процент ложноотрицательных результатов, что обусловлено коротким периодом нахождения адреналина и норадреналина в крови и их скорой метаболизации.

Поиск более надежных лабораторных маркеров ФХЦ привел к необходимости измерения метаболитов катехоламинов, обладающих значительно большим периодом полувыведения [4]. Одним из самых распространенных анализов во всем мире стало определение экскреции ванилилминдальной кислоты (ВМК) с мочой. Многие годы данный анализ считался наиболее информативным для диагностики ФХЦ. Тем не менее к недостаткам данного исследования относится зависимость уровня ВМК от активности синаптических ганглиев, в которых широко представлена моноаминоксидаза (МАО), под влиянием которой проиcходит синтез ВМК. Так, например, в покое около 80% циркулирующего в крови 3,4-дигидроксифенилгликоля, одного из предшественников ВМК, образуется дезаминированием норадреналина из синаптических нервов.

Совершенствование методов лабораторной диагностики привело к возможности определения промежуточных метаболитов катехоламинов — их метилированных производных, называемых общим термином — метанефрины. Синтез метанефринов происходит под действием фермента катехол-О-метилтрансферазы (КОМТ), которая присутствует в опухоли и ряде других тканей, но отсутствует в симпатических ганглиях, в связи с чем данный метаболит считается специфическим маркером катехоламинпродуцирующих опухолей. Продукция метилированных производных ФХЦ происходит в постоянном режиме и не зависит от выбросов катехоламинов в кровь [5][6][7]. Производное адреналина называется метанефрин, метаболитом норадреналина является норметанефрин. Большая часть метанефринов после попадания в кровь конъюгируется с сульфатами, образуя связанную форму. Как конъюгированные, так и свободные метаболиты выводятся из организма с мочой [8].

На настоящий момент определение уровня метанефринов считается наиболее информативным методом лабораторной диагностики ФХЦ. Существуют разные варианты лабораторного определения метаболитов катехоламинов: в плазме крови или в моче, общего количества или только свободной формы, фракционированный анализ (отдельное измерение уровней метанефрина и норметанефрина) или нефракционированный.

## ЦЕЛЬ ИССЛЕДОВАНИЯ

Целью данного исследования являлось сравнение эффективности различных анализов на определение уровня метанефринов в диагностике феохромоцитом.

## МАТЕРИАЛЫ И МЕТОДЫ

## Место и время проведения исследования

Место проведения. Клиника высоких медицинских технологий им. Н.И. Пирогова СПбГУ.

Время исследования. С ноября 2007 по декабрь 2022 гг.

## Изучаемые популяции

Пациенты, подвергшиеся хирургическому лечению новообразований надпочечников в указанный период.

Критерии включения. Пациенты с новообразованиями надпочечников с исследованным уровнем метанефринов крови или мочи.

Критерии исключения: наличие оперативных вмешательств по поводу образований надпочечников в анамнезе.

## Способ формирования выборки из изучаемой популяции

Формирование выборки сплошным способом.

## Дизайн исследования:

## Методы

В исследуемой выборке проведено сравнение четырех видов анализов на метанефрины: свободные фракционированные метанефрины плазмы, нефракционированные метанефрины суточной мочи, фракционированные общие метанефрины суточной мочи и фракционированные свободные метанефрины суточной мочи. Анализы были сданы пациентами амбулаторно в различных клиниках. Определение уровней метанефринов плазмы крови осуществлялось использованием иммуноферментного анализа (ИФА), суточной мочи — высокоэффективной жидкостной хроматографии (ВЭЖХ) на анализаторах полуавтоматического типа. Оценка значимости степени повышения уровня метанефринов проводилась путем исследования чувствительности и специфичности двукратного повышения от верхней границы нормальных значений. Кроме того, исследовалось наличие корреляции между уровнем метанефринов и размерами феохромоцитомы. В связи с тем, что анализы сдавались в разных лабораториях с различными референсными интервалами и единицами измерения, для унификации результатов определялся показатель, равный соотношению самого высокого результата анализа на верхнюю границу нормы для данного исследования. Полученный коэффициент демонстрировал степень повышения метанефринов и использовался для корреляционного анализа. Для расчета данного коэффициента при анализах фракционированных метанефринов использовался результат наиболее повышенного метаболита (метанефрина или норметанефрина). Для всех включенных больных характер новообразований надпочечников был подтвержден гистологически.

## Статистический анализ

Для статистического анализа использовались Microsoft excel и SPSS Statistics 23. Наличие корреляции между уровнем метанефринов и размером опухоли (неправильное распределение) оценивалось с использованием непараметрического критерия: коэффициента ранговой корреляции Спирмена. Значение p<0,05 считалось статистически значимым. Чувствительность и специфичность различных анализов высчитывалась с использованием сводных таблиц в Microsoft Excel.

## Этическая экспертиза

Заключение комитета по биомедицинской этике Клиники высоких медицинских технологий им. Н.И. Пирогова СПбГУ вх. №121 от 01.06.2023: учитывая ретроспективный характер исследования и отсутствие персональных идентификационных данных, неинтервенционный дизайн исследования, письменного согласия пациентов и специального одобрения этическим комитетом не требуется.

## РЕЗУЛЬТАТЫ

Всего обследованы 1088 пациентов, из которых 283 мужчины и 805 женщин в возрасте от 12 до 83 лет (49,6±13,3 года). В 348 случаях удаленная опухоль являлась ФХЦ, в остальных новообразования были представлены другими как гормон-секретирующими (кортикостеромы, альдостеромы, андростеромы), так и гормонально-неактивными новообразованиями надпочечников.

Полученные результаты оценки анализов на определение уровня метанефринов представлены в таблице 1. Исследование уровня свободных фракционированных метанефринов плазмы выполнило 232 пациента, нефракционированных метанефринов суточной мочи — 431 пациент, фракционированных общих метанефринов суточной мочи — 427 пациентов, фракционированных свободных метанефринов суточной мочи — 178. Анализ крови на свободные метанефрины плазмы показал наибольшую чувствительность с минимальным числом ложноотрицательных результатов. Наиболее специфичным оказался тест на нефракционированные метанефрины суточной мочи, с наименьшим числом ложноположительных результатов, однако данный тест показал низкий уровень чувствительности.

**Table:** Таблица 1. Результаты исследования эффективности используемых методов определения уровня метанефринов плазмы крови и суточной мочи в диагностике феохромоцитом Table 1. Results of a study of the effectiveness of the methods used to determine the level of metanephrines in blood plasma and 24-hour urine in the diagnosis of pheochromocytomas

	Чувствительность	Специфичность	Точность	Пациентов	Чувствительность при размере 3 см и менее	При оценке 2-кратного превышения нормы
Чувствительность	Специфичность	Точность
Фр. Свободные метанефрины плазмы	95,4%	90,3%	92,2%	232	96,6% (n=29)	84%	98%	92,70%
Нефр. Метанефрины мочи	67,60%	97,80%	90,70%	471	60,7% (n=28)	50,50%	99,20%	87,70%
Фр. Общие метанефрины мочи	92%	92,40%	92,30%	427	83,6% (n=55)	75,50%	98,90%	91,30%
Фр. Свободные метанефрины мочи	80,80%	90,50%	86,50%	178	75% (n=24)	65,80%	94,30%	82,60%

При исследовании было обнаружено, что наибольшее число ложноотрицательных результатов приходится на феохромоцитомы небольших размеров: 3 см и менее. При выполнении всех типов анализов мочи на метанефрины в данной группе чувствительность была ниже, чем среди опухолей всех размеров, за исключением анализа свободных метанефринов плазмы крови.

При оценке результата теста как положительного лишь при как минимум двухкратном повышении уровня метанефринов от верхней границы нормы специфичность всех тестов резко возросла, но произошло и ожидаемое снижение чувствительности. Так, для свободных метанефринов плазмы чувствительность снизилась с 95,4 до 84%, однако специфичность выросла с 90,3 до 98%.

При корреляционном анализе установлено, что между выраженностью повышения метанефринов, измеренным любым из четырех описанных тестов, и размером феохромоцитомы имелась положительная статистически значимая связь (табл. 2, рис. 1).

У пяти пациентов с феохромоцитомами, помимо повышения метанефринов, было также выявлено повышение уровня дофамина (у двух — по результатам анализа крови, у трех — суточной мочи).

**Table:** Таблица 2. Оценка наличия корреляции между размером опухоли и степенью повышения уровня метанефринов, измеренного различными методами Table 2. Assessment of the presence of correlation between tumor size and the degree of increase in metanephrine levels measured by various methods

Анализ на определение уровня метанефринов	Свободные метанефрины плазмы	Свободные метанефрины мочи	Общие фракционированные метанефрины мочи	Общие нефракционированные метанефрины мочи
Результаты оценки коэффицента корреляции	К. Спирмена 0,395 (p=0,001, N — 70)	К. Спирмена 0,533 (p=0,000005, N — 67)	К. Спирмена 0,315 (p=0,000259, N — 130)	К. Спирмена 0,227 (p=0,018, N — 108)

**Figure:**
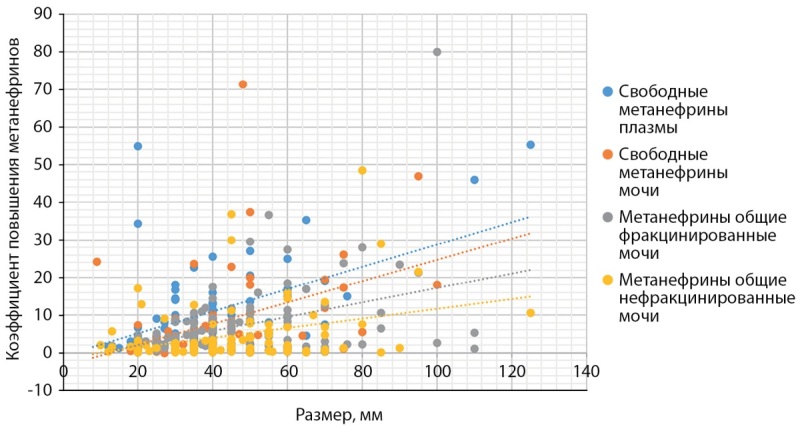
Рисунок 1. Корреляция между размером феохромоцитом и степенью повышения уровня метанефринов. Figure 1. Correlation between the size of pheochromocytomas and the degree of increase in metanephrine levels. *При оценке анализов на фракционированные метанефрины коэффициент рассчитывался по повышенному метаболиту, т.е. при нормальном уровне норметанефрина и повышенном уровне метанефрина учитывалась кратность повышения последнего.

## ОБСУЖДЕНИЕ

Так называемые общие метанефрины состоят из связанной и свободной фракций. Свободные метанефрины под действием фермента сульфотрансферазы, представленного преимущественно в гастроинтестинальном тракте, превращаются в сульфат-конъюгированные (связанные) производные [5]. Учитывая, что источником конъюгированных метанефринов являются те же свободные метанефрины, можно предположить, что тесты на определение свободных или общих метанефринов должны обладать сходными показателями чувствительности и специфичности. Однако норадреналин, продуцируемый в гастроинтестинальном тракте, оказывает большее влияние на развитие именно связанной фракции норметанефрина, и этот влияние может быть вариабельным. Кроме того, связанная и свободная фракции имеют разные периоды полувыведения. Свободные метанефрины быстро исключаются из циркуляции, метаболизируясь с помощью моноаминоксидазы или сульфотрансферазы или, не метаболизируясь, в небольших количествах выводятся с мочой. В то время как конъюгированные производные дольше сохраняются в кровотоке и почти полностью выводятся почками, в связи с чем их концентрация может резко увеличиваться при нарушениях почечной функции, а концентрация свободных метанефринов меньше подвержена данному влиянию [9][10].

Существующими клиническими рекомендациями для первичной лабораторной диагностики феохромоцитом рекомендовано определение свободных метанефринов плазмы или фракционированных метанефринов суточной мочи [11][12]. Оценке эффективности различных анализов на измерение уровня метанефринов плазмы и суточной мочи посвящено немало научных исследований [7][13][14][15]. Результаты их, однако, не всегда совпадают [14]. Lenders J.W.M. и соавт. в статье 1995 г. описывают результаты исследований, согласно которым измерение содержания метанефринов плазмы крови показало большую чувствительность, чем измерение метанефринов мочи или катехоламинов плазмы [4]. Eisenhofer G. и соавт. (2018 г.) в своем исследовании на 2056 пациентах установили более высокую чувствительность тестов на определение свободных метанефринов в плазме по сравнению с определением как свободных, так и общих метанефринов мочи, особенно в группе пациентов с высокой предтестовой вероятностью феохромоцитомы. Специфичность тестов на определение свободных метанефринов плазмы и свободных метанефринов мочи значимо не отличалась и была выше специфичности теста на определение общих (деконъюгированных) метанефринов мочи [15]. В статье по консенсусу рабочей группы по эндокринной гипертензии Европейского общества гипертоников, в авторах которой числятся оба исследователя, рекомендованными тестами первой линии называют исследования свободных метанефринов плазмы или мочи со схожей чувствительностью и специфичностью [1].

В целом, именно свободные метанефрины плазмы в большинстве случаев называют наиболее эффективным методом. Что подтверждалось и в нашем исследовании наиболее высокой чувствительностью метода, особенно при феохромоцитомах небольших размеров, а именно чувствительность и как следствие — высокая отрицательная прогностическая ценность является наиболее важной характеристикой теста для первичной диагностики [16]. Специфичность при исследовании свободных метанефринов плазмы крови была не самой высокой, однако она составляла приемлемые 90,3%, а при двукратном повышении доходила до 98%. Нефракционированные метанефрины мочи показали наихудший результат по чувствительности в диагностике феохромоцитом, но в тоже время обладали наибольшей специфичностью. В исследовании Lenders J.W.M. и соавт. (2002 г.) нефракционированные метанефрины мочи также обладали наибольшей специфичностью среди других тестов на определение данного метаболита катехоламинов. Этот метод, помимо низкой чувствительности, еще и не дает полезной в клинической практике информации о том, какой именно катехоламин повышен, в связи с чем, несмотря на высокую специфичность, является наименее предпочтительным тестом для первичной диагностики катехоламин-синтезирующих опухолей. Исследование фракционированных метанефринов мочи обладало хорошей чувствительностью и специфичностью и также является хорошим вариантом тестирования больных для диагностики гиперпродукции катехоламинов. Анализ суточной мочи на содержание свободных метанефринов неожиданно показал низкую эффективность в нашем исследовании, что не соответствует работам других авторов. Так, Lenders J.W.M. и соавт. (2017 г.) в своем исследовании описывают чувствительность и специфичность для свободных метанефринов мочи, скоректированных по креатинину в 97,2 и 98,1% соответственно [4]. Трудно судить, с чем может быть связана низкая эффективность этого анализа в нашем исследовании, однако следует отметить, что не было контроля по уровню креатинина, а также в обследованной группе пациентов данный анализ выполнялся существенно реже других (всего 178 пациентов), и, возможно, для получения более точных результатов требуется большее число случаев.

Для оценки вероятности наличия феохромоцитомы, помимо факта превышения верхнего порога нормальных значений, часто оценивается кратность его превышения [5][17][18]. Мы оценили показатели чувствительности и специфичности тестов при наличии более чем 2-кратного повышения уровня, что привело к ожидаемому снижению чувствительности и повышению специфичности результатов тестов. Наиболее заметным было увеличение специфичности с 90 до 98% для свободных метанефринов плазмы крови, что подтверждает важность учета степени повышения уровня метанефринов в клинической практике.

Обнаруженная корреляция между уровнем повышения метанефринов и размерами феохромоцитомы также подтверждается другими исследованиями [19][20]. Несмотря на статистически значимую корреляцию для всех видов анализов сила связи была умеренной или слабой. Это связано с тем, что размер опухоли не единственный фактор, влияющий на ее гормональную активность. Так, некрозы и кистозная дегенерация (нередко встречающиеся у крупных феохромоцитом), а также различия в гормональном метаболизме могут оказывать существенное влияние на уровень метанефринов, продуцируемый опухолью [20]. Изучение выраженности влияния данных факторов может быть направлением дальнейших исследований в данном вопросе.

На эффективность методов также существенное влияние может оказывать правильность сбора анализов. Для плазмы крови важным условием является забор крови в положении лежа после 30-минутного горизонтального положения, а при исследовании суточной мочи рекомендовано определение креатинина мочи для подтверждения полноты суточного сбора [11]. Отсутствие данных о качестве сбора анализов является значимым ограничением в интерпретации результатов данного исследования. Наиболее перспективным направлением дальнейшего развития данного исследования будет изучение большего числа случаев с учетом данных о качестве сбора анализов.

У пациентов с феохромоцитомами и повышенным уровнем дофамина в плазме крови или суточной моче каких-либо закономерностей по характеру течения артериальной гипертензии, размерам опухоли и степенью повышения метанефринов выявлено не было. У двух из них было установлено наличие наследственных синдромов: нейрофиброматоза 1 типа и синдрома множественной эндокринной неоплазии 2а типа.

## ЗАКЛЮЧЕНИЕ

Наиболее чувствительным методом диагностики феохромоцитом является исследование свободных метанефринов плазмы крови. Он демонстрирует высокую чувствительность даже при опухолях небольших размеров, а при значении, более чем в 2 раза превышающем верхнюю границу нормы, обладает и высокой специфичностью. Анализ на фракционированные общие метанефрины суточной мочи является немного менее чувствительным и более специфичным методом и также может эффективно применяться для первичной диагностики феохромоцитом. Между уровнем метанефринов, определенным любым из исследованных способов, и размерами феохромоцитом имеется статистически значимая положительная корреляционная связь.

## ДОПОЛНИТЕЛЬНАЯ ИНФОРМАЦИЯ

Источники финансирования. Работа выполнена по инициативе авторов без привлечения финансирования.

Конфликт интересов. Авторы декларируют отсутствие явных и потенциальных конфликтов интересов, связанных с содержанием настоящей статьи.

Участие авторов. Реброва Д.В., Краснов Л.М., Слепцов И.В. — идея и дизайн исследования; Федоров Е.А., Чинчук И.К., Черников Р.А. — предоставление материалов исследования; Реброва Д.В., Шихмагомедов Ш.Ш., Згода Е.А. — сбор данных, формирование выборки пациентов; Шихмагомедов Ш.Ш., Реброва Д.В. — формирование и ведение базы данных; Шихмагомедов Ш.Ш., Реброва Д.В., Русаков В.Ф., Слепцов И.В. — анализ и интерпритация данных, написание текста рукописи; Реброва Д.В., Русаков В.Ф., Краснов Л.М. — финальный анализ, редактирование текста рукописи.

Все авторы одобрили финальную версию статьи перед публикацией, выразили согласие нести ответственность за все аспекты работы, подразумевающую надлежащее изучение и решение вопросов, связанных с точностью или добросовестностью любой части работы.
